# Natural killer cells in inflammatory autoimmune diseases

**DOI:** 10.1002/cti2.1250

**Published:** 2021-02-01

**Authors:** Yuyan Yang, Jessica Day, Fernando Souza‐Fonseca Guimaraes, Ian P Wicks, Cynthia Louis

**Affiliations:** ^1^ Tsinghua University School of Medicine Beijing China; ^2^ Inflammation Division The Walter and Eliza Hall Institute of Medical Research Parkville VIC Australia; ^3^ Medical Biology University of Melbourne Melbourne VIC Australia; ^4^ Rheumatology Unit The Royal Melbourne Hospital Parkville VIC Australia; ^5^ University of Queensland Diamantina Institute Woolloongabba QLD Australia

**Keywords:** natural killer cells, autoimmune disease, rheumatoid arthritis, multiple sclerosis, systemic lupus erythematosus, type 1 diabetes mellitus, idiopathic inflammatory myopathies

## Abstract

Natural killer (NK) cells are a specialised population of innate lymphoid cells (ILCs) that help control local immune responses. Through natural cytotoxicity, production of cytokines and chemokines, and migratory capacity, NK cells play a vital immunoregulatory role in the initiation and chronicity of inflammatory and autoimmune responses. Our understanding of their functional differences and contributions in disease settings is evolving owing to new genetic and functional murine proof‐of‐concept studies. Here, we summarise current understanding of NK cells in several classic autoimmune disorders, particularly in rheumatoid arthritis (RA), multiple sclerosis (MS), systemic lupus erythematosus (SLE) and type 1 diabetes mellitus (T1DM), but also less understood diseases such as idiopathic inflammatory myopathies (IIMs). A better understanding of how NK cells contribute to these autoimmune disorders may pave the way for NK cell‐targeted therapeutics.

## Graphical abstract

In this review, we summarise current understanding of NK cells in several classic autoimmune disorders, particularly in rheumatoid arthritis (RA), multiple sclerosis (MS), systemic lupus erythematosus (SLE) and type 1 diabetes mellitus (T1DM), but also less understood diseases such as idiopathic inflammatory myopathies. We also discuss how a better understanding of the contribution of NK cells to these autoimmune disorders may pave the way for NK cell‐targeted therapeutics.

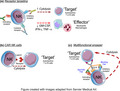

## Introduction

Autoimmune diseases are caused by inappropriate reactivity of adaptive immune cells towards self‐antigens and comprise highly heterogenous conditions. These diseases can be organ‐specific (i.e. multiple sclerosis and type 1 diabetes) or systemic (i.e. rheumatoid arthritis, systemic lupus erythematosus, and idiopathic inflammatory myopathies). While a complex interplay of genetic and environmental factors is thought to give rise to these discrete conditions, aberrant immune and inflammatory responses drive their initiation, progression and chronicity. Autoreactive T cells and autoantibody‐producing B cells (plasma cells) are key upstream drivers of autoimmune diseases.^1^, ^2^ Innate immune cells such as neutrophils, monocytes and macrophages are well‐described effector cells mediating tissue damage and inflammation in targeted organs.^3^, ^4^ However, other innate effector cells, including natural killer (NK) cells and other innate lymphoid cell (ILC) subsets, are also found in inflamed tissues and may regulate immune dysfunction and inflammation.^5^

NK cells are bone marrow‐derived granular cells with classic lymphoid cell morphology.^6^ The steady‐state NK cell reservoir includes blood, secondary lymphoid organs (spleen, lymph nodes and tonsils) and non‐lymphoid tissues (i.e. liver, uterus). In addition to the active recruitment of circulating NK cells, NK cells in non‐lymphoid organs (e.g. liver, skin, kidney and intestine) comprise a subset of tissue‐resident NK cells that develop from local precursors.^7^ Regardless of their origin, NK cells are geared for rapid modulation of immune responses.^7^

The ontogeny of NK cells has been extensively investigated. NK cells are generated from common lymphoid progenitors (which also give rise to T cells, B cells and other ILC subsets). They, however, do not rely on the thymic reservoir, but rather derive from bone marrow haemopoiesis and are dependent on the cytokine IL‐15.^8^, ^9^ NK cells and ILCs also differ from other lymphoid cells as they do not express somatically rearranged receptors and thus lack antigen specificity.^6^ In contrast, NK cells possess both cytotoxicity and cytokine‐producing properties, resembling CD8^+^ T cells and CD4^+^ T helper (Th) cells, respectively, and share similar transcription factor dependency (i.e. T‐bet and Eomes).^10^ Through cytotoxicity, cytokine production and migratory capacity, NK cells represent a highly versatile immune subset and contribute to many physiological and pathological settings, including protection against intracellular pathogens, anti‐tumor immunity, maternal‐foetal immune tolerance, graft rejection, chronic autoinflammatory diseases and autoimmune disorders.^11^, ^12^, ^13^, ^14^, ^15^ While the functional contributions of NK cells in infection and malignancy are clear, there have been mixed and often contradictory findings in autoimmune disease settings (discussed below). These discrepancies may relate to the different tissue niche or source of NK cells analysed, variable experimental systems, stage of disease, as well as the intrinsic disease heterogeneity. Here, we review current knowledge of NK cell biology in some major autoimmune diseases and discuss the future immunotherapeutic potential of NK cells.

## NK cell subsets

In humans, NK cells are commonly defined as CD3^–^ CD56^+^ NKp46^+^ mononuclear cells, which can be further classified into CD56^dim^ or CD56^bright^ subsets.^16^, ^17^ In mice, NK cells lack CD56 expression. Murine NK cells are commonly defined as CD3^–^ NK1.1^+^ NKp46^+^ CD49b^+^, and are further classified CD11b^+^ CD27^–^ and CD11b^–^ CD27^+^ subsets.^18^, ^19^, ^20^ High‐dimensional single‐cell analysis confirms that the two murine NK subsets represent conserved counterparts of human NK cell subsets.^17^

The CD56^dim^ NK cells are predominantly found in the blood and represent a highly cytotoxic subset. They express high levels of inhibitory killer immunoglobulin‐like receptors (KIRs), components of cytolytic granules (perforin and granzymes), and FcγRIIIA (CD16a^+^), which collectively mediate antibody‐dependent cell‐dependent cytotoxicity (ADCC).^6^, ^21^, ^22^ In contrast, the CD56^bright^ NK cells are abundant in secondary lymphoid tissues such as lymph nodes and tonsils,^21^, ^23^ in inflamed tissues and in the decidua during pregnancy.^24^, ^25^, ^26^ CD56^bright^ NK cells express lower levels of KIRs, cytolytic granules, and CD16 than CD56^dim^ NK subset, but have higher levels of cytokine receptors and inhibitory receptor CD94/NKG2A. Correspondingly, the CD56^bright^ NK subset is less cytotoxic but more efficient at cytokine and chemokine production than the CD56^dim^ subset.^21^, ^27^ CD56^dim^ and CD56^bright^ subsets also express discrete cytokine and chemokine receptors, which contribute to their temporal regulation in discrete niche.^21^

It should be noted that the cytolytic or cytokine‐producing capacity of NK cells is not necessarily restricted to a specific subset. For example, presumed cytokine‐producing CD56^bright^ NK cells may acquire cytolytic activity equal to, if not stronger than, CD56^dim^ NK cells upon appropriate stimulation through cytokines or activating receptors.^28^, ^29^ Conversely, CD56^dim^ NK cells can also produce IFN‐γ upon contact with target cells.^22^ Although cell‐surface markers can aid in identifying NK cell subsets in various anatomical locations, this does not necessarily define the functional phenotype of NK cells in different physiological settings. Furthermore, the bilateral system with CD56 as a classical means to separate NK subpopulations is becoming outdated. Both single‐cell transcriptomic and mass cytometric analyses have revealed remarkable phenotypic diversity of NK cell subpopulations in human peripheral blood and in primary tumors.^30^, ^31^, ^32^

## NK cell activation and licensing

In the steady state, peripheral NK cells are relatively quiescent but can rapidly respond to an array of germline encoded activating and inhibitory cell‐surface receptors. Sensing and signal integration downstream of these receptors allow NK cells to discriminate ‘altered self’ from ‘normal self’ and thereby play a critical role in host defence, while also keeping the potentially self‐destructive activity of NK cells in check.^33^ Examples of NK‐activating receptors include the KIR S family (or the corresponding murine homolog Ly49D and Ly49H), FcγRIII, CD94‐NKG2C complex, NKG2D, NKp46, IgG‐like receptor 2B4/CD244, adhesion molecules DNAM‐1/CD226, lymphocyte function‐associated antigen (LFA‐1), as well as various cytokine and chemokine receptors.^33^ Cytokines that regulate NK cell function include IL‐15, IL‐18, IL‐12, IL‐23, and type I interferons (IFNs).^34^, ^35^, ^36^, ^37^, ^38^, ^39^, ^40^, ^41^

Inhibitory NK cell receptors that recognise either classical or non‐classical major histocompatibility complex class I (MHC‐I) proteins balance NK‐activating signals. Example of NK‐inhibiting receptors include the human KIR L family (or the corresponding murine homolog Ly49A and Ly49C), CD94‐NKG2A complex, and TIGIT.^33^ According to the ‘missing self‐hypothesis', ubiquitously expressed MHC‐I ligands on host cells spares normal host cells from NK cell‐mediated lysis, while the loss or downregulated level of MHC‐I in infected or transformed cells renders host cells ‘foreign’ and thus susceptible to NK‐mediated killing.^42^ Paradoxically, the engagement of host MHC‐I with inhibitory receptors is also necessary for NK cells to gain full functional competence (NK cell licensing).^42^ Without such signals, unlicensed NK cells from MHC‐I‐deficient mice demonstrate diminished cytolytic and cytokine‐producing functions relative to NK cells from MHC‐I‐sufficient hosts.^43^, ^44^, ^45^, ^46^

NK cells are tightly controlled to prevent inappropriate responses against host cells.^33^ In keeping with the importance of fine‐tuning NK cells in immune homeostasis, several autoinflammatory conditions have been associated with mutations in NK cell activating receptors.^14^ NK‐activating and ‐inhibitory receptors can also be harnessed therapeutically. For example, blockade of the inhibitory NKG2A receptor promotes anti‐tumor immunity by enhancing the cytotoxic effector function of NK cells and CD8^+^ T cells.^47^ More recently, a first‐in‐class trifunctional agonistic antibody targeting two activating receptors (NKp46 and CD16‐mediated ADCC) and a tumor antigen on cancer cells augmented NK‐mediated tumor killing in preclinical murine models of cancer.^48^

Certain combinations of KIR and HLA molecules may predispose individuals to, or protect them from, inflammatory diseases. The expression of KIR S family members (e.g. KIR2DS1, KIR2DS2 and KIR3DS1), particularly when paired with certain HLA ligands, is associated with increased susceptibility to psoriatic arthritis,^49^, ^50^, ^51^ rheumatoid vasculitis,^52^ and SLE.^53^ However, these reports were observational and further functional studies are warranted to establish the disease‐promoting role of these KIR S isoforms in NK cells across autoimmune disorders. It is important to note that a lower level of NK inhibitory receptors does not necessarily translate to heightened autoreactivity. Rather, these ‘unlicensed’ NK cells may be rendered hyporesponsive to further stimulation by activating receptors. Conversely, elevated levels of inhibitory receptors could contribute to potent ‘licensing’ effects in NK cells in inflammatory settings. Indeed, patients with ulcerative colitis,^54^ Crohn’s disease^55^ and spondyloarthropathy^56^, ^57^ express more inhibitory KIRs (i.e. KIR2DL2/3 and KIR3DL1/2) and their cognate ligands compared with healthy controls. Spondyloarthropathy is associated with HLA‐B27,^56^, ^57^ tends to form heavy chain homodimers (so called B27_2_). B27_2_ can in turn interact with KIR3DL2 expressed by NK cells and T cells. Such interactions were shown to promote the survival and effector functions of KIR3DL2‐expressing NK cells and T cells, including enhanced NK cell cytotoxicity and T cell production of IL‐17.^56^, ^57^ Furthermore, genetically ‘licensed’ individuals, determined by the presence of KIR2DL2/3 and homozygosity for HLA‐C1, exhibit augmented CD4^+^ T cell proliferation and Th17 differentiation, compared with those from unlicensed individuals.^58^

## Immune regulatory functions of NK cells

Natural killer cells are implicated in both protective and pathogenic immunity, depending on the type and stage of the immune response, the target organ and NK subsets analysed. A well‐recognised function of NK cells is contact‐dependent cytotoxicity. This occurs through release of cytolytic granules containing proteases, granzymes, and perforins into target cells via a lytic synapse, and through the induction of caspase‐dependent apoptosis of target cells via the engagement of natural cytotoxicity receptors (NCRs), NKG2D, CD16a, LFA‐1, DNAM‐1, FAS ligands, and TNF‐related apoptosis‐inducing ligand (TRAIL).^28^, ^29^, ^48^, ^59^, ^60^, ^61^, ^62^, ^63^ Rapid NK cell‐mediated killing of infected or transformed cells is an important protective function against intracellular pathogens and malignancy, respectively.^64^ Self‐directed attack by cytotoxic NK cells could also aggravate pathology as has been observed in experimental autoimmune encephalomyelitis (EAE).^65^, ^66^ NK cells can also modulate immune responses by directly killing other immune cells such as monocytes/macrophages,^67^, ^68^, ^69^, ^70^ neutrophils,^59^, ^71^ and CD4^+^ Th17 and T follicular helper (Tfh) cell subsets.^29^, ^61^, ^63^, ^72^, ^73^, ^74^, ^75^, ^76^

Natural killer cells secrete cytokines and chemokines, orchestrating interactions with other immune cells. For example, NK cell‐derived IFN‐γ is essential for early Th1 priming in draining lymph nodes.^77^ Abundant IFN‐γ production by NK cells has also been associated with the pathogenesis of several inflammatory disorders such as SLE^78^, ^79^ and psoriasis.^80^ Furthermore, NK cells found in target non‐lymphoid tissues, such as the inflamed joint of RA patients,^81^, ^82^ the central nervous system (CNS) of MS patients,^83^ and the inflamed skin of psoriasis patients,^80^ were found to produce high levels of pro‐inflammatory cytokines including IFN‐γ and tumor necrosis factor‐α (TNF‐α). In addition to IFN‐γ and TNF‐α, NK cells may also secrete other cytokines such as granulocyte/macrophage‐colony stimulating factor (GM‐CSF), macrophage‐colony stimulating factor (M‐CSF), IL‐5, IL‐10, IL‐13, and chemokines CCL3, CCL4, CCL5, IL‐8, RANTES and XCL1.^83^, ^84^, ^85^, ^86^, ^87^, ^88^, ^89^, ^90^, ^91^, ^92^

The protective effect of NK cells in autoimmune disorders is thought to occur by the downregulation of autoreactive adaptive immune responses. NK cell ‘degeneration’, defined as numerical and/or functional deficits (reduced cytotoxicity), has been extensively documented in peripheral blood of patients with many inflammatory autoimmune diseases including RA,^93^ MS,^83^ T1DM,^94^ SLE,^95^ Sjogren’s syndrome^96^ and idiopathic inflammatory myopathies.^97^, ^98^, ^99^ These findings have also been observed in animal models of autoimmune diseases such as the collagen‐induced arthritis model for RA,^100^ a model of systemic juvenile idiopathic arthritis^69^ and the non‐obese diabetes (NOD) model for T1DM.^101^, ^102^ Reduced cytotoxicity of NK cells in these settings is thought to impair the restraint of pathogenic immune cells, while reduced circulating NK cell numbers might reflect enhanced recruitment to sites of inflammation. Enrichment of NK cells has been found in the synovial joints of RA patients,^25^, ^26^, ^81^ the kidneys of patients with lupus nephritis,^103^ cerebrospinal fluid and brain lesions of MS patients,^83^, ^104^ acute psoriatic plaques,^80^ and fibrotic lungs of patients with anti‐synthetase syndrome.^98^ These tissue NK cells have upregulated expression of tissue‐homing chemokine receptors.^26^, ^80^, ^81^, ^103^

Natural killer cell ‘degeneration’ does not necessarily portend autoimmunity. NK‐deficient (*Mcl1^fl/fl^*:Ncr1Cre) mice,^105^ or patients with congenital deficiency in NK cells,^106^ do not develop spontaneous autoimmunity, most likely because of compensatory tolerogenic mechanisms and immune checkpoints. Studies using anti‐NK1.1 or anti‐asialoGM1 monoclonal antibodies to deplete NK cells in mice have produced mixed results (discussed below). In the following sections we discuss how NK cells, which only account for a small fraction of total lymphocytes, nevertheless contribute to the outcomes of inflammatory and autoimmune diseases.

## NK cells in rheumatoid arthritis

Rheumatoid arthritis (RA) is a chronic inflammatory autoimmune disease that is characterised by persistent joint inflammation, cartilage damage, and bone erosion.^107^ Studies using the autoimmune collagen‐induced arthritis (CIA) model of RA identify Th17 cells and germinal centre‐dependent humoral responses as key drivers of disease,^108^, ^109^, ^110^ while innate immune cells (neutrophils, monocytes/macrophages and NK cells), fibroblast‐like synovial cells and bone‐resorbing osteoclasts cause joint inflammation and destruction.^111^, ^112^, ^113^, ^114^

In RA, the frequency of NK cells is increased in peripheral blood of patients^81^, ^115^ but these NK cells consistently display impaired effector functions such as reduced IFN‐γ production and decreased cytotoxicity.^115^, ^116^, ^117^, ^118^ This may be due to upregulation of inhibitory receptors such as CD161^117^ and NKG2A,^119^ or downregulation of activating receptors such as CD16.^116^ Deficient cytotoxic function of peripheral NK cells is thought to contribute to the early phase of autoimmune arthritis. Robust CIA induction was observed following adoptive transfer of collagen type II‐specific CD4^+^ T cells and B cells into *Rag2^–/–^ Prf1^–/–^* hosts (lacking T and B cells, and perforin‐deficient NK cells), but not into *Rag2^–/–^* hosts (lacking T and B cells, but with perforin‐sufficient NK cells).^119^ Although *ex vivo* experiments demonstrated NK‐mediated lysis of arthritogenic Th17 and Tfh cells,^119^ this may not reflect a physiological scenario because other immune checkpoints (i.e. T regulatory cells) are absent. IFN‐γ produced by NK cells is also thought to inhibit arthritis, both in the passive transfer autoantibody‐induced arthritis^120^ and the autoimmune CIA^100^ models. However, NK‐mediated inhibition of passive transfer autoantibody‐induced arthritis is only apparent following CpG‐oligonucleotide stimulation.^120^ In the CIA model, NK‐derived IFN‐γ is thought to limit Th17 differentiation as NK cells depletion with anti‐asialoGM1 at priming phase led to the expansion of Th17 cells and mild exacerbation of CIA at disease onset.^100^ A more sustained exacerbation of CIA was similarly observed following anti‐NK1.1‐mediated depletion, but this appears to be due to NK T cell depletion as CD1d^–/–^ (NK T‐deficient) mice also develop worse CIA.^121^

**Figure 1:**
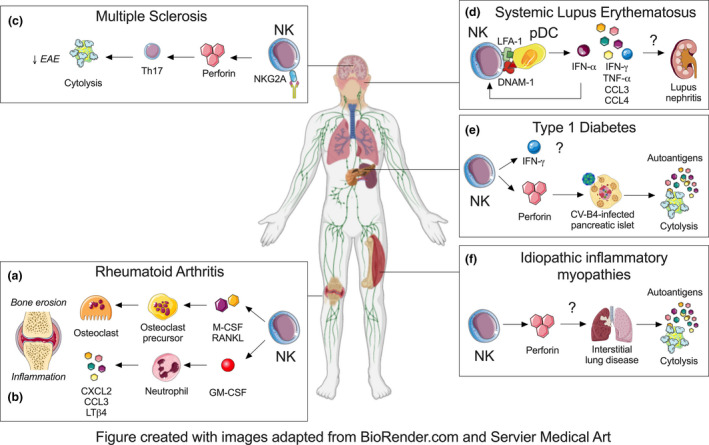
NK cell involvement in autoimmune inflammatory diseases. NK cells exacerbate RA by secreting soluble mediators such as **(a)** M‐CSF and RANKL that drive the differentiation of bone‐eroding osteoclasts and **(b)** GM‐CSF that promotes the production of pro‐inflammatory mediators by joint‐infiltrating neutrophils. **(c)** NK cells do not appear to play a dominant role in MS but boosting their cytotoxic function with anti‐NKG2A may eliminate encephalitogenic Th17 cells and alleviate disease in the EAE model. **(d)** NK cells may promote SLE through their interaction with pDCs via LFA‐1 and DNAM‐1 that enhances the production of cytokines and chemokines such as IFN‐α, IFN‐γ, TNF‐α, IL‐6, IL‐8, CCL3 and CCL4. NK cells are also found in kidney of lupus nephritis patients but it remains unclear if NK cells and their cytokine dysfunction contribute to tissue pathology. **(e)** NK cells could contribute to the generation of autoantigens through excessive killing of CV‐B4‐infected pancreatic β islets. However, other functions of NK cells such as IFN‐γ production remain unclear and future studies are required to capture phenotypic and functional diversity of NK cells in both CV‐B4‐associated and sterile T1DM subtypes. **(f)** Alveolar NK cells are thought to give rise to autoantigens such as histidyl tRNA synthetase following respiratory insults in anti‐synthetase syndrome. Future studies are needed to evaluate whether similar numerical and functional changes in NK cells occur in the discrete subtype of IIM.

Abundant NK cells are present in RA synovium and most harbour a unique CD56^bright^ phenotype.^25^, ^26^, ^115^, ^122^ RA synovial NK cells are CD69^+^ NKp44^+^, indicative of their activated state, but are perforin^low^.^25^, ^26^, ^115^, ^122^ They also upregulate surface expression of inhibitory CD94‐NKG2A,^123^ which strongly inhibits NK cell production of IFN‐γ and TNF and also restrains cytotoxicity upon binding to its ligand, HLA‐E.^25^, ^119^, ^122^ Unlike circulating NK cells, synovial NK cells have low KIR expression,^25^, ^26^, ^81^ but express chemokine receptors, such as CCR5, CXCR3 and CCR1, which may facilitate their preferential recruitment into RA synovium.^26^, ^81^ Given the low cytotoxicity and IFN‐γ production of synovial NK cells,^115^ these NK cells likely contribute to local joint inflammation by producing other pro‐inflammatory mediators. Reciprocal activation of joint‐infiltrating CD56^bright^ NK cells and CD14^+^ inflammatory monocytes has also been suggested in RA.^25^, ^124^ Murine studies identify joint NK cells as sources of M‐CSF and RANKL that promote the differentiation of bone‐resorbing osteoclasts^89^ (Figure 1a). In contrast to earlier studies,^100^ the depletion of NK cells using anti‐asialoGM1 attenuated both joint inflammation and bone erosion in the CIA model.^89^ These studies demonstrate the limitations of antibody depletion of NK,^125^, ^126^ which can be further confounded by the dynamics of autoimmune responses.

Others suggest additional mechanisms by which NK cells can regulate local joint inflammation. For example, IL‐15‐activated NK cells trigger apoptosis of mature bone‐resorbing osteoclasts via LFA‐1, DNAM‐1 and TRAIL.^127^ Another study demonstrates the degranulation of an NK cell line in response to RA fibroblast‐like synoviocytes, upon stimulation via NKG2D, DNAM‐1, NKp46 and NKp44 receptors.^123^ These studies, however, were limited to *in vitro* observations using blood NK cells and monocyte‐derived osteoclasts^127^ or an NK cell line^123^ and contradict reports showing low cytotoxicity of synovial NK cells.^115^, ^116^, ^117^, ^118^ Synovial NK‐derived IFN‐γ has also been thought to limit arthritis by inhibiting Th17 polarisation in the CIA model,^100^ but this seems questionable given the low IFN‐γ production by RA synovial NK cells.^115^ More recently, we reported that synovial joint NK cells propagate joint inflammation by secreting the pro‐inflammatory cytokine GM‐CSF^91^ (Figure 1b). This occurs in an IL‐18‐dependent manner, and independently of their cytotoxic function or IFN‐γ production.^91^ GM‐CSF, in turn, signals to joint‐infiltrating myeloid cells such as neutrophils to upregulate pro‐inflammatory CXCL2, CCL3 and LTβ4, thereby sustaining immune cell recruitment into inflamed joints.^91^ To our knowledge, this was the first study to define how joint NK cells participate in local inflammatory cascades in the effector phase of autoantibody‐induced arthritis.

In summary, synovial NK cells and peripheral NK cells may perform distinct immune functions in RA. Current evidence shows that synovial NK cells aggravate arthritis through the production inflammatory mediators such as GM‐CSF, M‐CSF and RANKL, thereby priming effector myeloid cells. In contrast, peripheral NK cells in secondary lymphoid tissues may exert a protective role in RA by virtue of IFN‐γ production and cytotoxicity against arthritogenic immune cells. Future studies should carefully consider the possibility of inherent differences in NK cells at anatomical locations. Better understanding of the NK‐activating and ‐inhibitory repertoire in RA could inform NK‐based therapies.

## NK cells in multiple sclerosis

Multiple sclerosis (MS) is an inflammatory neurodegenerative disease characterised by autoreactive T cell‐induced demyelination of the CNS, leading to progressive neurological deficits.^128^ Experimental autoimmune encephalomyelitis (EAE) is a widely used model for MS and is induced by active immunisation or passive transfer of myelin oligodendrocyte glycoprotein antigen‐specific T cells.^129^ Disease induction in both MS patients and EAE model is driven by Th17 cells, particularly the GM‐CSF‐producing subset,^130^, ^131^, ^132^ and to some extent by autoantibodies.^133^

In relapsing‐remitting MS, a reduction in peripheral NK cell activity was found to coincide with clinical relapse.^134^ *Ex vivo* cytokine‐activated NK cells demonstrate potent lytic capacity towards autologous CD4^+^ T cells following the engagement of surface receptors including NKp30, NKp46, DNAM‐1, NKG2D, LFA‐1 and TRAIL.^28^, ^29^, ^61^, ^83^ Peripheral NK cells from untreated MS patients, however, were less efficient in suppressing autologous CD4^+^ T cells compared with healthy controls.^29^, ^83^ Compromised cytotoxicity in MS NK cells may result from upregulated T cell expression of HLA‐E, the ligand for the inhibitory receptor NKG2A,^29^ or downregulated DNAM‐1/CD155‐mediated NK cell priming.^83^ Elevated levels of HLA‐E have also been detected in the cerebrospinal fluid and CNS plaques of MS patients, which correlated with reduced NK cytotoxicity and higher disease activity.^135^ Collectively, these studies suggest compromised NK‐mediated removal of myelin‐reactive T cells could contribute to MS. This functional deficiency might reflect resistance of autoreactive T cells to the cytolytic activity of NK cells, rather than an intrinsic NK cell defect.

The involvement of NK cell in MS remains contentious. NK cell depletion with anti‐NK.1.1 antibody exacerbated murine EAE.^65^, ^72^ Similarly, mice deficient in the CX3CR1 chemokine receptor, which directs NK cell recruitment to the CNS, showed worse EAE, while inducible NK cell expansion with IL‐2 attenuated EAE.^65^ In these settings, the protective role for NK cells in EAE development is thought to be mediated by CNS‐recruited NK cells and to occur through indirect suppression of Th17 responses via microglia^65^ or killing of CNS‐infiltrating CCR2^+^ Ly6C^+^ inflammatory monocytes.^68^ In support of the notion of protective effects for NK cells in MS, treatment with biological disease‐modifying agents,^136^, ^137^, ^138^ is associated with restoration of circulating NK cell number. However, these studies are limited to correlative observations and may not reveal the true regulatory functions of NK cells.

In an earlier study, NK cell depletion with either anti‐NK.1. or anti‐asialoGM1 was shown to reduce EAE severity.^139^ These discrepancies might be explained by differences in immunisation and antibody dose. In a more recent and comprehensive report, passive transfer of encephalitogenic 2D2 transgenic Th17 cells into mice treated with anti‐NK1.1 antibody, or into mice with NKp46‐lineage specific depletion of T‐bet (*Tbx21^fl/fl^*:NKp46Cre), resulted in significantly reduced EAE. This finding underscores the importance of NK1.1^+^ NKp46^+^ ILCs (i.e. NK cells, ILC1 and/or ILC3 subsets).^140^ Further, passive transfer of encephalitogenic 2D2 Th17 cells induced EAE of comparable incidence and severity in NK‐sufficient and NK‐deficient (*Eomes^fl/fl^*:NKp46Cre) mice.^140^

Taken together, NK cells do not appear to have a significant physiological role in Th17‐induced autoimmune neuroinflammation. Nevertheless, manipulation of NK cell function through inhibitory or activating receptors may still offer a viable therapeutic strategy in MS. For example, pharmacological blockade of the inhibitory NKG2A or its ligands Qa‐1, was shown to enhance NK‐mediated killing of autoreactive CD4^+^ T cells, skew Th1/Th17 responses towards a non‐pathogenic Th2 response, and ameliorate EAE^73^, ^141^ (Figure 1c).

## NK cells in systemic lupus erythematosus

Systemic Lupus Erythematosus (SLE) is a chronic, systemic autoimmune disease and substantial clinical heterogeneity makes it one of the most therapeutically challenging autoimmune disorder.^142^ Hallmarks of SLE include hyperactivation of type I IFN responses, sustained production of multiple autoantibodies against nuclear autoantigens, and immune complex formation in various organs (i.e. skin, kidney, lung, blood, joint, and CNS), leading to tissue inflammation and damage, such as lupus nephritis. In addition to autoimmune T and B cells, plasmacytoid dendritic cells (pDCs) play a prominent role in lupus through type I IFN production, namely IFN‐α, downstream of innate immune recognition of self‐DNA and ‐RNA through toll‐like receptors (TLRs).^143^, ^144^

In SLE, both the absolute number and frequency of NK cells are diminished in the peripheral blood of patients, especially in those with active disease or with severe clinical manifestations such as lupus nephritis and thrombocytopenia.^95^, ^145^, ^146^, ^147^ Some studies found no difference in the proportions of NK cell subsets,^78^, ^145^ while others observed an increased frequency of CD56^bright^ NK cells.^148^ These discrepancies might be explained by immunosuppressive therapies the patients were receiving. Phenotypic alterations in SLE NK cells include increased expression of CD69, NKp46, CD86, and OX40/CD134,^78^, ^146^, ^148^, ^149^ suggesting a dysfunctional state. CD69 expression on NK cells appears to correlate with disease activity.^149^ Other changes include upregulation of inhibitory CD94‐NKG2A and reduced CD16.^78^ However, the expression of other NK receptors, including NKG2C, NKG2D and KIR family members, is less definitive, likely reflecting clinical heterogeneity of SLE.^78^, ^149^, ^150^

NK cells might contribute to protection against SLE by eliminating DCs,^146^, ^150^ but peripheral NK cell cytotoxicity is impaired in SLE patients irrespective of disease activity.^95^, ^145^, ^146^, ^148^, ^149^, ^151^ This impaired cytotoxicity might, at least partially, result from an intrinsic NK cell defect, because first‐degree relatives of SLE patients showed similar impairment of NK‐mediated killing compared with healthy donors.^151^ Alternative explanations include inhibition by anti‐lymphocyte antibodies,^95^, ^152^ reduced IL‐2Rβ expression on NK cells,^95^ and/or a defective NK response to IL‐15.^149^, ^153^ Autoantibodies to CD94 and KIRs have been described in SLE and may contribute to the reduced cytotoxic function of NK cells and increase the risk of lupus nephritis.^154^, ^155^

Natural killer cells have been implicated in the pathogenesis of SLE through their interactions with pDCs.^150^, ^156^, ^157^ NK cells augment IFN‐α production by immune complex‐activated pDCs through the secretion of CCL4 and cell–cell interaction in an LFA‐1‐ and DNAM‐1‐dependent manner^156^ (Figure 1d). In turn, pDC‐derived IFN‐α is essential for NK cell development, maturation and IFN‐γ production.^150^, ^158^ Bidirectional activation of these two cell types establishes a highly inflammatory milieu containing abundant cytokines and chemokines, including IFN‐α, IFN‐γ, TNF‐α, IL‐6, IL‐8, CCL3 and CCL4^157^, ^159^ (Figure 1d). Other murine studies support a pathogenic role for NK cells in lupus. In the TLR7 transgenic and FcγRIIB^–/–^ murine models of SLE, chronic TLR7 signalling has been associated with extended survival of NK cells and proliferation of immature NK cells.^146^, ^160^ An atypical NK cell subset that possesses both NK‐ and DC‐like functions has also been reported.^160^ These unique NK/DC co‐express NK1.1, CD11c, CD122 and MHC‐II, respond to IL‐15 stimulation, produce type I and II IFNs, are highly proliferative, and demonstrate both cytotoxic and antigen‐presenting functions. Remarkably, adoptive transfer of these atypical NK/DC cells to wildtype mice induces lupus‐like autoimmunity.^160^ Consistent with this finding, a similar subset of CD3^–^ CD56^+^ HLA‐DR^+^ CD11c^+^ NK cells have been identified in SLE patients, although their precise role remains to be defined.^146^ In another study,^147^ a subset of proliferating Ki67^+^ NK cells were identified in SLE patients and was associated with more severe disease, active nephritis and a lowered total NK cell number. Whether these HLA‐DR^+^ CD11c^+^ or Ki67^+^ NK cells truly represent the human counterpart of pathogenic murine NK:DCs in SLE remains to be determined.^147^, ^160^ More recently, inducible expansion of cytotoxic lymphocytes with an IL‐15 superagonist led to the exacerbation of lupus parameters in mice. However, this was shown to be driven primarily by CD8^+^ T cell expansion and not NK cells,^161^ arguing against a direct pathogenic role of NK cells in lupus, at least in the MRL/lpr lupus model.

Lupus nephritis is a serious manifestation of SLE and is often studied as a model for organ involvement. Single‐cell RNA sequencing revealed an abundance of both CD56^dim^ CD16^+^ and CD56^bright^ CD16^–^ NK subsets in kidney biopsies of patients with lupus nephritis,^103^ but what role these NK cells play has not been characterised (Figure 1d). Similarly, in murine MRL/lpr and MRL/MpJ models of SLE, NK cells are actively recruited into kidneys during the early phases of disease.^79^, ^150^ These observational studies await functional evidence to confirm the involvement of NK cells in the pathogenesis of SLE.

In summary, interaction of peripheral NK cells with pDCs in a reciprocal manner could contribute to an exaggerated systemic inflammatory response in SLE, that is characterised by type I and type II IFN responses. Further investigations are needed to determine if interfering such NK‐pDC crosstalk is therapeutically beneficial and to define if organ‐infiltrating NK cells play an immune regulatory function in lupus nephritis.

## NK cells in type 1 diabetes mellitus

Type 1 diabetes mellitus (T1DM) is probably mediated by CD8^+^ T cells, which selectively destroy pancreatic β cells, causing insulin deficiency and hyperglycaemia.^162^ Inflamed islets are infiltrated by cytotoxic CD8^+^ T cells. CD4^+^ T cells, B cells, and NK cells have also been found.^163^, ^164^, ^165^

Studies characterising NK cell numbers and activity in human T1DM demonstrate that NK cell deficiency is universal. A decrease in peripheral blood NK cell number was observed both in patients with newly onset T1DM^94^ and those with long‐standing disease.^166^ Furthermore, functional deficiencies of NK cells in long‐standing diabetic patients have been reported. Although blood NK cells displayed a hyperactivated state (IFN‐γ‐producing) at disease onset, these cells expressed lower levels of activating receptors (i.e. NKG2D, NKp30 and NKp46) and had decreased IFN‐γ expression and NKG2D‐dependent cytolytic activity in the chronic phase of disease, compared with control subjects.^94^, ^166^

A disease‐promoting effect of NK cells in diabetes is proposed based on studies in the NOD mice. NK cells infiltrate into the pancreas prior to T cells and have an activated phenotype, with enhanced proliferation and spontaneous IFN‐γ production and degranulation^101^, ^102^, ^167^ (Figure 1e). The proportion and numbers of NK cell infiltrating the pancreas positively correlated with autoimmune responses in NOD mice, and depletion of NK cells significantly inhibited anti‐CTLA‐4‐induced exacerbation of diabetes.^168^ In this study, early islet destruction was proposed to be due to NK cell‐derived IFN‐γ.^168^ However, transgenic NOD mice expressing a dominant negative IFN‐γ receptor on β cells had a similar incidence of diabetes compared to non‐transgenic mice,^169^ indicating that any direct effect of IFN‐γ on β cells is dispensable for diabetes, at least in the NOD model.

Abundant NK cell infiltration and degranulation were reported during the evolution of destructive insulitis in low‐dose streptozotocin‐induced diabetes model.^167^ Genetic deletion or blockade of NKp46 abrogated the development of diabetes in NOD mice. A subsequent study, however, queried the role of NK cells in spontaneous diabetes in NOD mice because anti‐NK1.1‐induced NK cell depletion caused only a slight delay in the onset of full‐blown disease.^102^

Interestingly, destruction of pancreatic β cells by NK cells and the subsequent development of T1DM can occur following enterovirus infection. Animal and human studies have highlighted the role of NK cells in mediating group B4 coxsackieviruses (CV‐B4)‐induced autoimmunity against islet cells.^164^, ^170^ In chronic infection, CV‐B4‐infected islet cells can downregulate the surface expression of HLA class I molecules on β cells and avoid recognition and killing by cytotoxic T cells. These islets remain susceptible to NK cell‐mediated elimination^170^ but β cell apoptosis could lead to the release of potential autoantigens that may trigger autoreactivity and cause further β cell destruction^171^ (Figure 1e). Consistently, NK cell depletion using anti‐asialo‐GM1 reduced early CVB4‐induced insulitis and islet destruction in SOCS1‐Tg NOD mice.^170^ NK cells may therefore contribute to CV‐B4‐associated T1DM through excessive killing of infected pancreatic β islet cells.

In sum, current studies suggest that NK cells could contribute to the generation of autoantigens in enterovirus‐associated T1DM through pancreatic β islet killing. However, the role of NK cells in sterile T1DM is unknown. Future studies are required to capture phenotypic and functional diversity of NK cells in both CV‐B4‐associated and sterile T1DM subtypes.

## NK cells in idiopathic inflammatory myopathies

Idiopathic inflammatory myopathies (IIMs) comprise a group of uncommon chronic inflammatory autoimmune diseases affecting skeletal muscles. IIMs have been further classified into several subtypes based on differences in immunopathology, including polymyositis, dermatomyositis, anti‐synthetase syndrome, immune‐mediated necrotising myopathy, sporadic inclusion body myositis and nonspecific myositis.^172^ IIMs are characterised by chronic muscle inflammation and destruction, leading to muscle fibre degeneration and weakness. The causes of IIMs are unclear but autoreactive CD8^+^ T cells, CD4^+^ T cells and/or autoantibodies have all been identified in muscle biopsies.^173^

As in other autoimmune disorders, reductions in the number and frequency of circulating NK cells have been observed in active dermatomyositis,^174^ and levels normalise with disease remission.^99^, ^175^ An increased number of NK cells have been reported in affected muscles of juvenile dermatomyositis patients early in the disease course,^176^ but not in adult dermatomyositis patients.^177^ Impaired cytotoxicity of NK cells has been reported in dermatomyositis, which may be related to compromised PLCγ2 signalling and a defect in calcium flux.^99^ Whether and how NK cells contribute to dermatomyositis remains unclear.

A role for NK cells in the pathogenesis of anti‐synthetase syndrome has been hypothesised, specifically in the generation of autoantigens (Figure 1f). Anti‐synthetase syndrome is defined by the presence of autoantibodies against tRNA synthetases, most commonly histidyl tRNA synthetase, and characteristic clinical features include myositis and extramuscular manifestations (i.e. interstitial lung disease and arthritis).^178^ Although anti‐synthetase syndrome is conceptualised as a myopathy, mounting evidence suggests disease induction likely occurs in the lungs. Epidemiologically, anti‐synthetase syndrome is strongly associated with prior respiratory insults,^179^, ^180^ and an immunogenic, granzyme B‐cleavable of histidyl tRNA synthetase has been identified in alveolar epithelium.^181^ While NK cells are scarce in the inflamed muscles of patients with anti‐synthetase syndrome, they are greatly expanded in affected lungs and express granzyme B.^98^ The number of circulating NK cells in active anti‐synthetase syndrome patients is comparable to healthy controls, but there is a higher frequency of NK cells expressing granzyme B. These NK cells display a mature CD57^hi^ phenotype but low levels of NKp30 activating receptor.^98^ This pattern appears to be specific to anti‐synthetase syndrome, as the percentage of differentiated CD57^+^ NK cells in the circulation of sporadic inclusion body myositis and immune‐mediated necrotising myopathy patients is comparable to healthy controls.^182^ Whether alveolar NK cells contribute to the initiation of autoimmunity through the generation of immunogenic peptides of histidyl tRNA synthetase in the anti‐synthetase syndrome warrants further investigation (Figure 1f).

Compared to other autoimmune disorders described above, NK cells remain less well defined in IIMs. At least in anti‐synthetase syndrome, alveolar NK cells are thought to give rise to autoantigens such as histidyl tRNA synthetase following respiratory insults. However, it is unknown whether this occurs through excessive cytolysis of alveolar epithelial cells and/or granzyme B‐mediated cleavage of histidyl tRNA synthetase. Future studies are needed to evaluate whether similar numerical and functional changes in NK cells occur in the discrete subtype of IIM.

## NK cell therapy in autoimmunity

**Figure 2:**
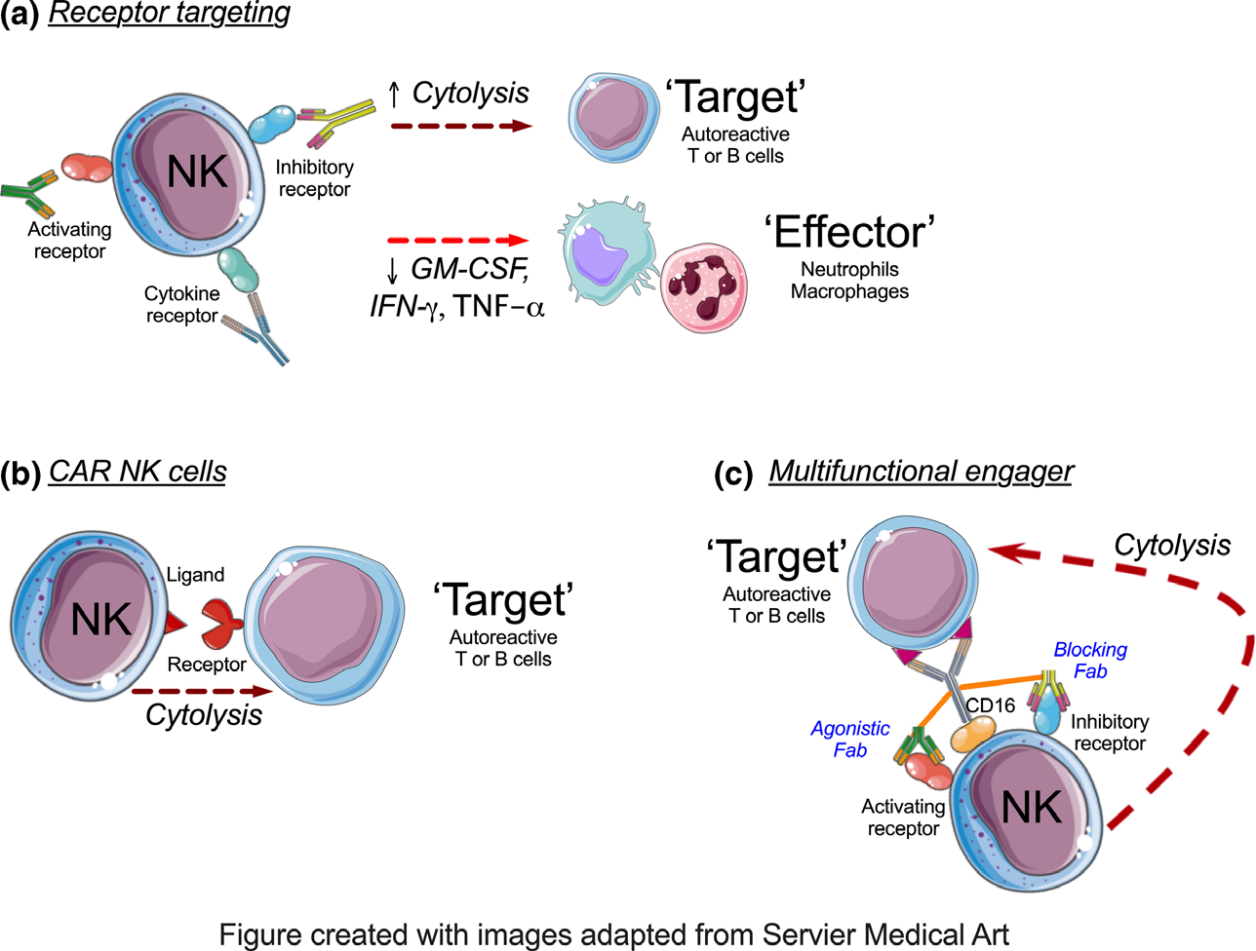
Therapeutic potential of NK‐based therapy in autoimmune disorders. **(a)** Blocking or agonistic antibodies target cell‐surface receptors of NK cells to potentiate their cytotoxicity against autoreactive immune cells, or suppress the production of pathogenic cytokines. **(b)** Engineering of chimeric antigen receptor (CAR) could direct NK cells to eliminate autoreactive immune cells. **(c)** A multifunctional engager combining antibodies that target antigens expressed by autoreactive cells and activating/inhibitory receptors on NK cells could facilitate NK cell interaction and cytolytic activity towards target autoimmune lymphocytes.

Natural killer cells may hold significant therapeutic potential in autoimmune diseases. First, blocking or agonistic antibodies that target activating, inhibitory and/or cytokine receptors could be used to potentiate cytotoxic activity against autoreactive immune cells, or to suppress the production of pathogenic cytokines such as GM‐CSF, IFN‐g and TNF‐α (Figure 2a). For example, anti‐NKG2A blocks an NK inhibitory receptor and potentiates NK cells cytotoxicity towards pathogenic Th17 and Tfh, alleviating EAE,^73^, ^141^ as well as CIA.^119^ Second, engineering of chimeric antigen receptor (CAR) NK cells could eliminate autoreactive immune cells in a ‘targeted’ manner (Figure 2b). This approach follows the use of CD19‐targeted CAR T cell therapy to deplete autoreactive B cells in murine lupus.^183^ Subsequently, CAR NK cells expressing PD‐L1 were shown to eliminate PD‐1^hi^ Tfh cells *ex vivo* and in a humanised mouse model of lupus‐like disease.^184^ Alternatively, engineering of a multifunctional engager that incorporates combination of antibodies targeting antigens expressed by autoreactive immune cells and activating/inhibitory receptors could facilitate NK cell interaction and cytolytic activity (Figure 2c). Multifunctional NK cell engagers have recently demonstrated for cancer immunotherapy.^48^

## Conclusions and perspectives

Considerable evidence suggests that numerical and/or functional deficits in NK cells are common in a variety of inflammatory autoimmune diseases. However, the mechanisms underpinning this abnormality may differ between diseases. It remains contentious whether NK cell cytotoxic function constitutes an immune checkpoint by direct elimination of autoreactive immune cells or is merely a consequence of disease. Recent appreciation of NK cells’ heterogeneity in cancer studies calls for a more careful interpretation of past studies in autoimmune disease settings. The integration of new technical advances such as single‐cell transcriptomic and mass cytometry may similarly reveal an underappreciated phenotypic diversity of NK cells in autoimmune diseases.

Studies defining the role of NK cells in cancer have paved the way for NK cell‐based cancer immunotherapies. We believe detailed understanding of the temporal and spatial functions of NK cells in autoimmune inflammatory diseases could similarly offer opportunities to target NK cells in concert with other immune‐modifying therapies. While several KIR‐HLA haplotypes have been genetically associated with discrete autoimmune disorders, caution should be exercised when drawing conclusions from observational and *ex vivo* studies using patient samples. These novel hypotheses must be accompanied by proof‐of‐principle experiments at functional protein level with genetic and pharmacologic *in vivo* models, such as NK‐specific conditional knockout mice or KIR isoform‐specific antibodies. Such information may also be relevant in understanding immunotherapy‐associated rheumatic adverse events, particularly with the increasing use of immune checkpoint inhibitors and introduction of NK‐based cancer immunotherapies. Whether NK cell dysfunction contributes to the development of these complex autoimmune‐like conditions merits further investigation.

## Conflict of Interest

FSFG is a consultant for Biotheus Inc.

## Author Contributions

**Yuyan Yang:** Writing‐original draft; Writing‐review & editing. **Jess Day:** Writing‐review & editing. **Fernando Souza‐Fonseca‐Guimaraes:** Writing‐review & editing. **Ian Wicks:** Writing‐review & editing. **Cynthia Louis:** Conceptualization; Supervision; Visualization; Writing‐original draft; Writing‐review & editing.
